# LetibotulinumtoxinA for Rosacea: A Pilot Study

**DOI:** 10.3390/toxins18040162

**Published:** 2026-03-28

**Authors:** Agnieszka Bańka-Wrona, Witold Wrona, Wioletta Barańska-Rybak

**Affiliations:** 1 Faculty of Medicine, Łazarski University, 02-662 Warsaw, Poland; 2 Rederm, 02-594 Warsaw, Poland; 3 HealthQuest Sp. z o.o., 01-517 Warsaw, Poland; 4 Faculty of Medicine, Medical University of Gdańsk, 80-210 Gdańsk, Poland

**Keywords:** botulinum toxin A, letibotulinumtoxinA, rosacea

## Abstract

Rosacea-associated erythema and flushing often remain inadequately controlled by standard therapies. Intradermal botulinum toxin A has emerged as a potential treatment targeting the neurovascular component of rosacea. This pilot study aimed to evaluate the safety and preliminary efficacy of intradermal letibotulinumtoxinA for persistent erythema and flushing in rosacea. Eleven patients with refractory erythematotelangiectatic rosacea received a single session of intradermal letibotulinumtoxinA (20 U total dose). Outcomes at 2 weeks included clinician- and patient-rated erythema severity, patient-reported flushing, skin hydration, sebum, elasticity, and Dermatology Life Quality Index (DLQI). Safety assessments included adverse events and pain. Two weeks post-treatment, 73% of patients showed improvement in Clinician’s Erythema Assessment score and 100% reported reduced flushing. Median hydration and elasticity increased, while the level of sebum decreased. Median DLQI improved from 9 to 2. No serious adverse effects occurred; mild, transient cheek heaviness and dryness were noted in three cases. Intradermal letibotulinumtoxinA was well tolerated, with few reported side effects/complications. The treatment demonstrated preliminary efficacy in reducing rosacea erythema and flushing, and improving skin physiology and quality of life; however, these require confirmation in a larger, controlled study.

## 1. Introduction

Rosacea is a common, chronic inflammatory dermatosis characterized by recurrent episodes of facial erythema, flushing, telangiectasia, and, in some cases, papules, pustules, or phymatous changes. It affects approximately 3–5% of the adult population globally [1,2] and is associated with a considerable psychosocial burden. Individuals with rosacea frequently experience reduced self-esteem and diminished quality of life (QoL), largely due to the persistent and visible nature of the disease [3,4]. Clinically, rosacea can be categorized into several subtypes [3]. Among them, erythematotelangiectatic rosacea (ETR), also known as vascular rosacea or type 1 rosacea, is one of the most prevalent and distressing forms [5].

The pathophysiology of rosacea is multifactorial and incompletely understood, involving a complex interplay between neurovascular dysregulation and chronic cutaneous inflammation. In ETR, transient receptor potential ion channels are overexpressed on peripheral sensory neurons and non-neuronal cells [6]. This, in response to external stimuli, may lead to the release of vasoactive neuropeptides, notably calcitonin gene–related peptide, substance P, and vasoactive intestinal peptide, which mediate vasodilation and contribute to the exaggerated facial flushing and persistent erythema characteristic of the disease [7]. In parallel, an aberrant innate immune response sustains cutaneous inflammation. Elevated levels of the antimicrobial peptide cathelicidin, observed in patients with rosacea, trigger pro-inflammatory and pro-angiogenic pathways in the skin [8]. Mast cells act as key amplifiers of this inflammatory response by releasing proteases and cytokines in response to neuroimmune signals [7,9]. The combined effects of these neurovascular and immune mechanisms establish a self-perpetuating cycle of vasodilation and inflammation, which underlies the persistent erythema observed in rosacea.

Managing ETR is challenging, as current therapies have significant limitations. Standard treatments such as topical metronidazole or azelaic acid and oral tetracycline-class antibiotics primarily target the inflammatory lesions and offer only modest relief for erythema and flushing [10,11,12]. Physical and pharmacological modalities that directly address vasodilation, including vascular laser or intense pulsed light therapy and topical α-adrenergic agonists such as brimonidine or oxymetazoline, can temporarily reduce facial redness, but their effects are often transient and can be associated with relapses or rebound phenomena [11,12]. In many patients with severe or refractory ETR, persistent erythema and episodic flushing remain inadequately controlled despite these interventions [10]. This therapeutic gap has prompted the exploration of novel treatment approaches targeting the neurovascular component of rosacea.

Emerging preclinical and clinical evidence support the therapeutic potential of botulinum toxin type A (BoNT-A) in the management of rosacea. In a murine model of rosacea-like dermatitis, pretreatment with onabotulinumtoxinA significantly attenuated LL-37–induced erythema, mast cell degranulation, and expression of inflammatory mediators, thereby demonstrating a mechanistic benefit of BoNT-A in modulating neurogenic inflammation [13]. Clinically, several prospective studies have reported symptomatic improvement following intradermal administration of BoNT-A [14,15,16,17,18,19,20,21]. A recent prospective trial in patients with treatment-resistant ETR demonstrated marked reductions in facial erythema and flushing, accompanied by significant improvements in QoL. The therapeutic effects were sustained for an average of 3 to 6 months post-treatment, with a low incidence of adverse events, underscoring the favorable safety profile of BoNT-A in this context [18]. A recent systematic review encompassing seven studies concluded that BoNT-A may be an effective, generally well-tolerated intervention for rosacea. The above findings support further investigation of BoNT-A as a promising therapy targeting both the vascular and inflammatory components of rosacea.

LetibotulinumtoxinA, a novel formulation of BoNT-A, has shown safety and efficacy comparable to those of established BoNT-A products for treatment of glabellar lines [22]. Its potential advantages include improved precision of action and reduced immunogenicity [23,24], which may offer particular benefits in the treatment of chronic inflammatory dermatoses such as rosacea. However, to date, its use in rosacea has not been investigated in clinical or preclinical settings, and its therapeutic utility in this context remains to be determined.

In this pilot study, we aimed to assess the efficacy, safety, and tolerability of intradermal injections of letibotulinumtoxinA in patients with ETR. The study was designed to address whether the treatment can reduce facial erythema and the frequency/severity of flushing episodes, and to evaluate its effects on objective skin parameters of rosacea severity and on patients’ quality of life.

## 2. Results

Of the 11 patients, 6 were women and 7 were men. Ten patients had ETR; 1 showed features of ETR and papulopustular rosacea. All patients had erythema inadequately controlled by standard therapies. Clinicians’ and patients’ assessment of rosacea varied in severity (Table 1). At baseline, flushing was present in the majority of patients.

All patients received the studied treatment and had all assessments at baseline and follow-up, except one man who did not complete the instrumental assessments.

### 2.1. Efficacy

**Figure 1:**
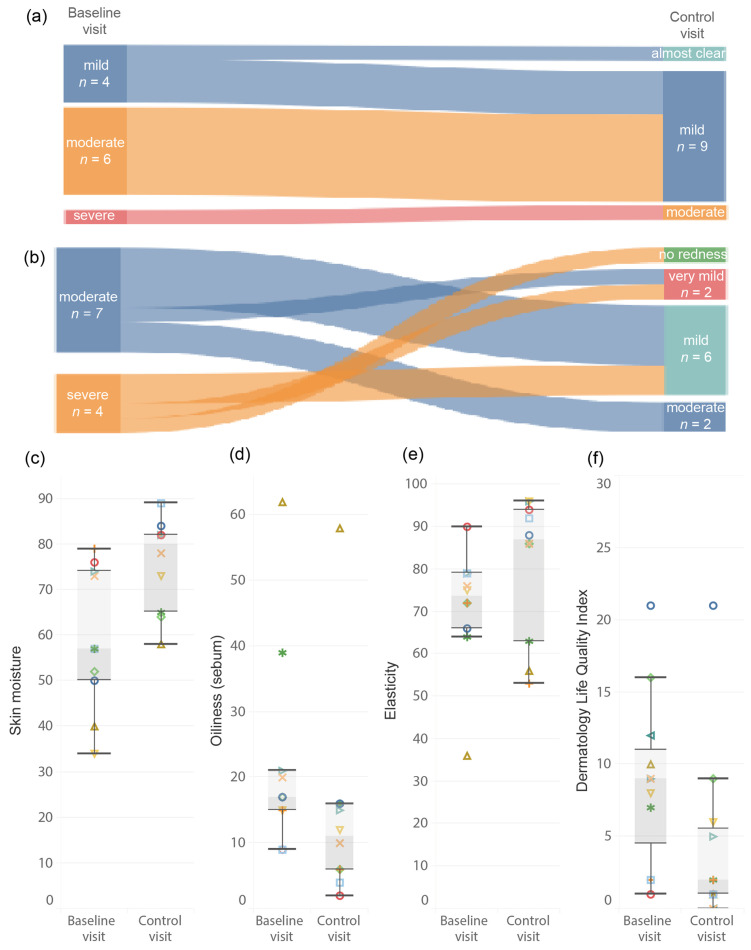
Efficacy outcomes. (**a**) Changes in Clinician’s Erythema Assessment scale (*n* = 11); (**b**) Changes in Patient’s Self-Assessment scale (*n* = 11). Changes in instrumental measurements, including (**c**) skin hydration, (**d**) sebum level, and (**e**) skin elasticity (*n* = 10). (**f**) Changes in Dermatology Life Quality Index (*n* = 11). On panels (**c**–**f**) color symbols indicate individual patients.

The median Clinician’s Erythema Assessment (CEA) score improved from 3 (range, 2–4) to 2 (1–3); *p* = 0.0047. The CEA scores improved by one category in patients with moderate and severe rosacea. Patients with mild rosacea (*n* = 4) did not improve, except one, who achieved almost clear skin; *p* = 0.0047 (Figure 1a). The overall improvement, evaluated by a physician, was observed in eight patients (73%). The median Patient’s Self-Assessment (PSA) improved from 3 (range, 3–4) to 2 (0–3); *p* = 0.0070. The score improved significantly in all patients with severe rosacea (*n* = 4); each patient declared improvement of ≥2 categories. Among patients with moderate PSA score (*n* = 7), two had no change (Figure 1b). The treatment had no negative effect on CEA and PSA scores. All patients who reported flushing at baseline reported a decrease in its intensity after treatment.

Objective evaluation of skin parameters two weeks after the treatment revealed improvement in skin characteristics. Median skin hydration changed from 57 (range, 34–79) to 80 (range, 58–89); *p* = 0.0038 (Figure 1c). Skin oiliness decreased from a median of 17 (range, 9–62) to 11 (range, 2–58); *p* = 0.0020 (Figure 1d). The median skin elasticity increased from 73.5 (range, 36–90) to 87 (53–96); *p* = 0.0488 (Figure 1e).

**Figure 2:**
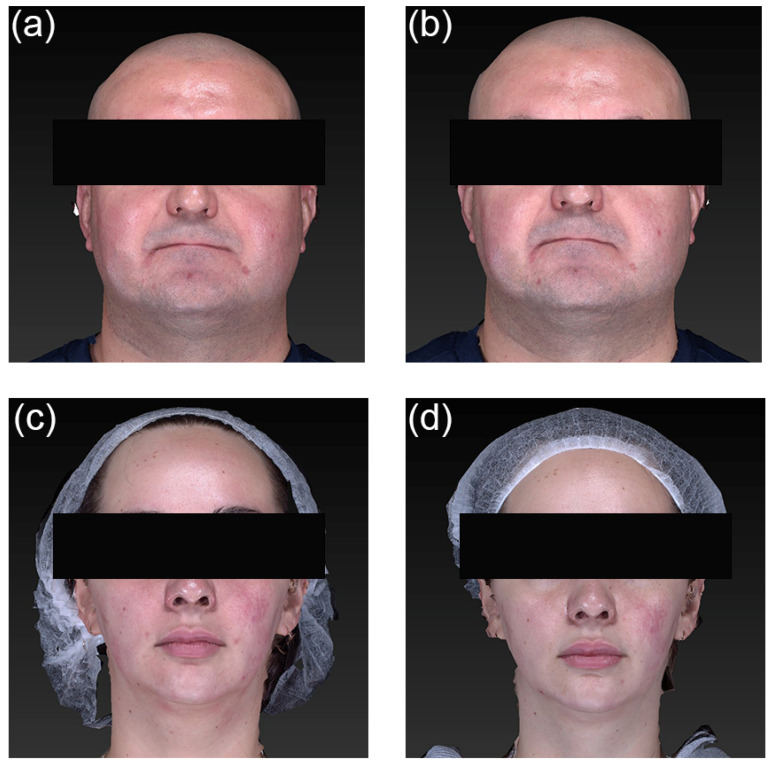
Representative cases of clinical photographs at baseline and two weeks after treatment. A male with erythematotelangiectatic rosacea (**a**) before and (**b**) after the treatment. A female with erythematotelangiectatic rosacea (**c**) before and (**d**) after the treatment.

The median Dermatology Life Quality Index (DLQI) score at baseline was 9 (range, 1–21), and at follow-up, it was 2 (0–21); *p =* 0.0116. A minimal clinically important difference of ≥4 points [25] was observed in 6 patients (55%). None of the patients reported an increase in disease burden. Representative facial photographs of the patients demonstrating improvements at each visit are shown in Figure 2.

### 2.2. Safety

The median pain intensity measured by the VAS was 7 (range, 2–8). None of the patients experienced immediate side effects. At follow-up, two patients reported heavy cheeks: one lateral and one bilateral. One patient reported dry skin, accompanied by a significant decrease in sebum (from 39 to 16).

## 3. Discussion

The results of this preliminary study demonstrate that intradermal letibotulinumtoxinA can significantly ameliorate the persistent erythema and flushing of rosacea, with corresponding improvements in patient-reported outcomes and skin physiology. The majority of patients showed a reduction in CEA, and all reported a decrease in PSA severity, along with meaningful reductions in flushing frequency and intensity. Overall QoL improved in parallel with clinical signs, underscoring the psychosocial benefit of reducing the visible symptoms of rosacea. Objective skin assessment indicated enhanced skin hydration and elasticity after treatment, accompanied by reduced sebum production. These findings support our working hypothesis that letibotulinumtoxinA’s neuromodulatory effects may interrupt the neurovascular dysregulation of rosacea, yielding both symptomatic relief and measurable dermal changes.

In the majority of patients treated with leibotulinumtoxinA, despite improvement, erythema persisted, as assessed by both the physician and the patients. The reason behind this can be a too low dose of toxin. Evidence suggests a dose–response relationship for erythema reduction. Comparison of intradermal microinjections of 0.5 U versus 1 U of BoNT-A per site revealed significantly greater improvements in facial erythema with the higher dose [20]. Specifically, the 1 U/point side showed more pronounced reductions in CEA scores between 2 and 8 weeks post-treatment and lower objective redness on imaging than the 0.5 U/point side. The potency of BoNT-As can not be directly compared. However, a total dose of 20 U of leibotulinumtoxinA may be at the lower end of the therapeutic spectrum, i.e., is sufficient to inhibit neurotransmitters that trigger episodic flushing but suboptimal for attenuating baseline vasodilation underlying persistent erythema.

The results obtained appear comparable to those reported for other BoNT-A formulations. Kim et al. [16] conducted a split-face, placebo-controlled trial in which one cheek was treated with intradermal prabotulinumtoxinA and the other with saline only. They also observed reductions in clinician erythema scores on toxin-treated sides and improvements in global esthetic ratings. The authors measured biophysical skin parameters up to 12 weeks after the treatment. Skin hydration and elasticity were significantly improved on toxin-treated skin, which mirrors the increases in hydration and elasticity documented in our cohort. Improved skin biophysical properties may reflect secondary benefits of reduced neurogenic inflammation, as chronic vasodilation and inflammation in rosacea are known to disrupt the epidermal barrier and dermal matrix [26]. It is worth noting that Kim et al. [16] did not find a significant change in sebum production on the toxin-treated side of the face. In contrast, our patients showed lower sebum levels post-treatment, suggesting a possible dose- or formulation-dependent effect. Early reports suggest that intradermal BoNT-A may reduce sebaceous gland activity. Shah [27] reported subjective reductions in sebum production and facial pore size following intradermal BoNT-A injections. It is unknown whether specific BoNT-A properties, dosing or injection technique lead to a more pronounced effect.

Our results contribute to a growing consensus that intradermal BoNT-A is a safe option for patients with rosacea, particularly those who do not respond satisfactorily to conventional therapies. The procedure is relatively painful. Previously, some groups have used topical anesthetics prior to the procedure [15,16,18]. All patients in our series tolerated the procedure well; facial tightness and dryness were the only reported adverse events, consistent with the favorable safety profile reported in other studies. Notably, none of our patients experienced unwanted muscle weakness, likely because of the use of microdoses and superficial placement. Using ≤0.02 mL of BoNT-A per site minimizes diffusion-related paresis [28].

High patient satisfaction rates in BoNT-A studies [29], including ours, indicate that appropriately selected patients perceive meaningful improvement in their appearance and symptoms after this therapy. Nonetheless, proper patient selection and counseling are key. Botulinum toxin does not directly address papules/pustules or telangiectatic vessels; therefore, it is best suited for the vascular-dominant (erythematotelangiectatic) phenotype or mixed phenotypes in which redness is a major complaint.

Our study has several limitations. First, this was a pilot study with a small sample size, a lack of a control group, and a relatively short follow-up period; it was not intended to demonstrate efficacy but to assess the direction of effects and safety. The improvements we observed, however, were consistent with other reports and could be partly attributable to the placebo effect or to spontaneous fluctuations in rosacea symptoms [30]. This requires elucidation in a controlled trial. Earlier studies comparing BoNT-A with placebo saline injections reported a reduction in erythema only in the treatment arm [31]. Second, the follow-up duration in our study was short. A two-week treatment-assessment interval is a minimal period when the effects of treatment can be observed in some studies [15,20]. While partial improvement can begin within the first 1–2 weeks, the peak effect on persistent erythema often manifests about 1–2 months post-injection [15]. Although objective and subjective improvement were observed, we cannot determine precisely when the therapeutic effect peaks or how long it ultimately lasts. Other studies suggest that the effect of a single BoNT-A treatment wanes by approximately 3–6 months [18,31]. Finally, the patient population was heterogeneous, with mild-to-severe ETRs and discrepancies between corresponding physician and patient assessments. These limitations underscore that our positive results should be interpreted as a proof of concept. Our finding needs confirmation in a larger, randomized, blinded study to reduce bias arising from the above-mentioned limitations.

## 4. Conclusions

In conclusion, this single-arm pilot study provides preliminary evidence that intradermal leibotulinumtoxinA can be safely used to treat ERT and may reduce erythema and flushing and improve quality of life. The observed effects require confirmation in a larger, controlled study.

## 5. Materials and Methods

This was a prospective study conducted at a single center in Poland. The study documentation was reviewed and approved by the ethics committee of the Lazarski University on 20 June 2023 (code: 12/KB/2023, Warsaw, Poland). All patients provided informed consent to participate in the study and separately consented to the publication of photographs taken during the study. Patients were recruited from April 2025 to August 2025.

### 5.1. Patients

Key inclusion criteria were age ≥ 18 years, diagnosis of ETR with persistent erythema, and willingness to comply with study procedures. Exclusion criteria included pregnancy or breastfeeding, any neuromuscular disorders, active infection or skin condition in the treatment area, recent use of systemic rosacea treatments (such as oral antibiotics, isotretinoin, or other photosensitizing or anti-rosacea agents), and need for any other emergent rosacea interventions. All participants provided written informed consent after a thorough explanation of the experimental treatment and its off-label nature. A preliminary assessment of each patient was conducted by a dermatologist at the baseline visit, and the medical history was completed.

### 5.2. Intervention

**Figure 3:**
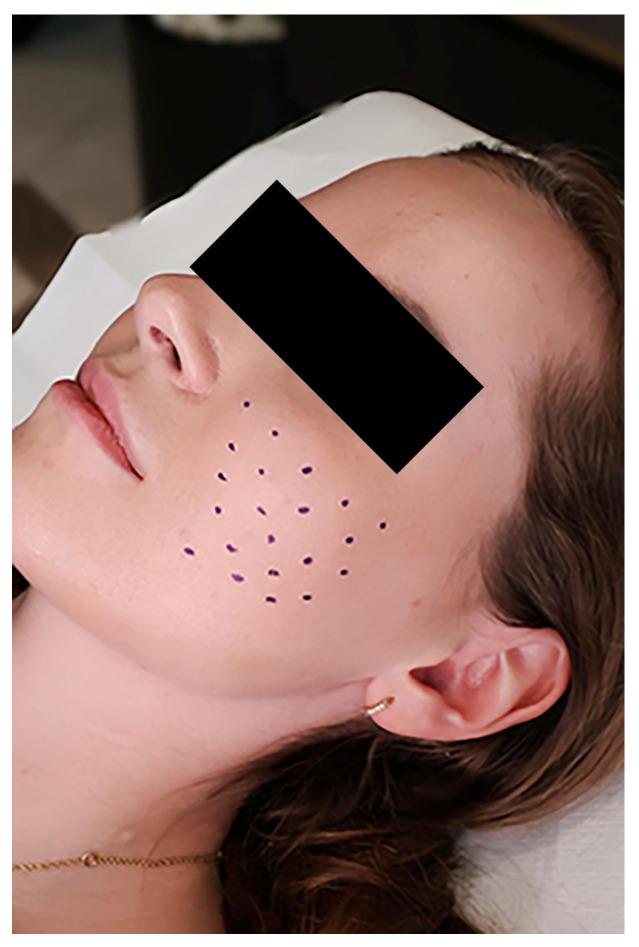
Sketch marking the injected area.

Each patient received a single treatment session of intradermal injections of letibobotulinumtoxinA (Letybo, Croma-Pharma GmbH, Leobendorf, Austria). The product was supplied in 50 U vials of lyophilized powder. For administration, the toxin was reconstituted with sterile 0.9% saline to a concentration of 20 U/mL. The injections were performed using a syringe fitted with a 30-gauge needle. The toxin was injected very superficially into the dermis of the affected facial areas (primarily the bilateral malar regions) using a microdroplet technique. Injection points were spaced approximately 1 cm apart over the regions of persistent erythema on each cheek. Each injection of 0.5 U (~0.025 mL) was administered intradermally to avoid diffusion into the facial muscles. The final dose was 20 U (10 U per side), administered in around 20 injections (Figure 3). No topical anesthesia was used before the injections. During the study period, patients were required to continue their routine topical rosacea treatments (e.g., metronidazole or azelaic acid) and basic skincare if these had been established prior to enrollment. However, initiation of any new systemic or topical therapy for rosacea or other procedural treatments was not permitted to isolate the effects of the botulinum toxin intervention.

### 5.3. Efficacy Assessments

Efficacy was evaluated by both clinical and patient grading and objective evaluation at baseline and at a follow-up visit 2 weeks after the injection. The primary efficacy endpoint was improvement in facial erythema severity, as assessed by the investigator using the CEA scale. The CEA is a 5-point ordinal grading of erythema, ranging from 0 (clear, no signs of erythema) to 4 (severe erythema) [32]. At each visit, patients and physicians independently graded overall central face erythema. The PSA scale was used as a key secondary endpoint, in which patients independently rated the severity of their rosacea redness on a 5-point scale (from none to severe) based on their own perception [33]. Patients also reported the frequency and intensity of transient flushing episodes qualitatively at each visit, and whether flushing had improved compared with baseline.

Objective skin parameter measurements were obtained using a multi-probe skin analysis device (Courage + Khazaka Electronics GmbH, Cologne, Germany) to provide quantitative data on skin physiology. Specifically, three parameters were measured on the cheek area: skin surface hydration, sebum level, and skin elasticity. Skin moisture was measured in arbitrary hydration units using the Corneometer^®^ probe (Courage + Khazaka Electronics GmbH, Cologne, Germany), which assesses the capacitive moisture content of the superficial skin. Sebum levels on the skin surface were measured using a Sebumeter^®^ probe;(Courage + Khazaka Electronics GmbH, Cologne, Germany), which quantifies sebum secretion via an optical grease-film method. Skin elasticity was assessed as a percentage or index by an elastometric suction device (Cutometer^®^ probe; Courage + Khazaka Electronics GmbH, Cologne, Germany), which measures the viscoelastic properties of the skin. All instrument measurements were taken in a controlled environment (room temperature and relative humidity) after patients had acclimated for at least 15 min, to ensure consistency. These instrumental assessments were recorded at baseline and at the 2-week follow-up visit to detect any physiological skin changes that might correlate with clinical changes.

In addition to erythema ratings, disease burden and quality of life were assessed using the DLQI questionnaire. The DLQI is a validated 10-item survey that yields a score from 0 to 30, with higher scores indicating greater impairment of quality of life due to skin disease [34]. The DLQI was completed by patients at baseline and again at the 2-week follow-up.

### 5.4. Safety Assessments

After the injection procedure, patients remained in the clinic for 15 min to observe for any immediate reactions. Local tolerability was assessed by asking patients to rate the injection-related pain using a visual analog scale (VAS) immediately after treatment (0 = no pain, 10 = worst pain imaginable). The treating physician examined the injection sites for acute reactions such as bleeding, swelling, or significant bruising. At the 2-week follow-up, all participants were interviewed and examined for adverse events, with specific attention to known potential effects of BoNT-A and any changes in skin condition.

### 5.5. Data Analysis

The analysis was primarily descriptive. No formal sample size calculation was performed a priori. The cohort size of approximately 15 patients was determined by the number of eligible patients available during the enrollment period. All collected data were entered into an electronic database. For ordinal scales, results were summarized as changes between categories. For continuous outcomes, descriptive statistics, such as the median and range, were used. The normality of the data was assessed using the Shapiro–Wilk test. Changes in assessed parameters were analyzed using either the paired t-test or the Wilcoxon test. Data was analyzed using MedCalc version 23.3.7 (MedCalc Software Ltd., Ostend, Belgium) and visualized using Tableau 2025.2.4 (Salesforce Inc., San Francisco, CA, USA).

## Figures and Tables

**Table 1:** Patient characteristics.

	N = 11
Female sex, *n* (%)	6 (55)
Age, median (range)	33.5 (26–47)
Fitzpatrick skin type, n (%)	
I	4 (36)
II	6 (55)
III	1 (9)
Past treatment, *n* (%)	
Metronidazol	6 (55)
EBD-based treatment	5 (45)
Ivermectin	5 (45)
Azelaic acid	3 (27)
Tetracyclines	3 (27)
Isotretinoin	2 (18)
Tacrolimus	1 (9)

EBD, energy-based device.

## Data Availability

The original contributions presented in this study are included in the article. Further inquiries can be directed to the corresponding author.

## Abbreviations

The following abbreviations are used in this manuscript:

BoNT-A	Botulinum toxin type A
CEA	Clinician’s Erythema Assessment
DLQI	Dermatology Life Quality Index
ERT	Erythematotelangiectatic rosacea
PSA	Patient’s Self-Assessment
QoL	Quality of life
VAS	Visual Analog Scale
